# Mimotope ELISA for Detection of Broad Spectrum Antibody against Avian H5N1 Influenza Virus

**DOI:** 10.1371/journal.pone.0024144

**Published:** 2011-09-02

**Authors:** Yingwei Chen, Wenxin Luo, Huijuan Song, Boyuan Yin, Jixian Tang, Yixin Chen, Mun Hon Ng, Anthony E. T. Yeo, Jun Zhang, Ningshao Xia

**Affiliations:** National Institute of Diagnostics and Vaccine Development in Infectious Diseases, The Key Laboratory of Education Minister for Cell Biology and Tumor Cell Engineering of Xiamen University, School of Life Sciences, Xiamen University, Xiamen, China; Wageningen University and Research Centre, The Netherlands

## Abstract

**Background:**

We have raised a panel of broad spectrum neutralizing monoclonal antibodies against the highly pathogenic H5N1 avian influenza virus, which neutralize the infectivity of, and afford protection against infection by, most of the major genetic groups of the virus evolved since 1997. Peptide mimics reactive with one of these broad spectrum H5N1 neutralizing antibodies, 8H5, were identified from random phage display libraries.

**Method:**

The amino acid residues of the most reactive 12mer peptide, p125 (DTPLTTAALRLV), were randomly substituted to improve its mimicry of the natural 8H5 epitope.

**Result:**

133 reactive peptides with unique amino acid sequences were identified from 5 sub-libraries of p125. Four residues (2,4,5.9) of the parental peptide were preserved among all the derived peptides and probably essential for 8H5 binding. These are interspersed among four other residues (1,3,8,10), which exhibit restricted substitution and probably could contribute to binding, and another four (6,7,11,12) which could be randomly substituted and probably are not essential for binding. One peptide, V-1b, derived by substituting 5 of the latter residues is the most reactive and has a binding constant of 3.16×10^−9^ M, which is 38 fold higher than the affinity of the parental p125. Immunoassay produced with this peptide is specifically reactive with 8H5 but not also the other related broad spectrum H5N1 avian influenza virus neutralizing antibodies. Serum samples from 29 chickens infected with H5N1 avian influenza virus gave a positive result by this assay and those from 12 uninfected animals gave a negative test result.

**Conclusion:**

The immunoassay produced with the 12 mer peptide,V1-b, is specific for the natural 8H5 epitope and can be used for detection of antibody against the broad spectrum neutralization site of H5N1 avian influenza virus.

## Introduction

As defined by conventional serology, the neutralization site of the influenza A virus encompasses the site located to the head of the HA molecule, where the virus contacts with host cells to initiate infection and area in the proximity of it, such that binding of antibody to this site arrests infection [1]. Antigenic specificity of the site changes rapidly, enabling the virus to evade host immune surveillance, thereby resulting in recurrent seasonal outbreaks and contributing to regular occurrence of influenza pandemics [2], [3]. Current effort to control the infection is to predict the antigenic specificity of the emerging strains on the basis of those circulating presently and in the past [4], [5]. The entailing difficulty is that the prediction is not always accurate and that vaccines might not be produced in time.

The recent discovery of a distinct type of broadly cross reacting and relatively conserved (BCRC) neutralization sites is significant, because they present an alternative and a more stable target to control the infection. Identified by monoclonal antibodies instead of conventional antisera, one of such neutralizing sites, designated broad spectrum H5N1 neutralizing site [6], [7], [8], is present in most of the major genetic groups (clades) of the H5N1 highly pathogenic avian influenza virus isolated since 1997, when the latter first re-emerged [2]. The other, the heterosubtypic neutralizing site, is present in different HA subtypes of influenza virus [8], [9], [10]. It is especially significant that co-crystalization of the heterosubtypic antibody and HA molecules has located the hetersubtypic neutralizing site to the stem of the HA molecule [11], [12], because this physically separates the newly identified neutralizing site from the neutralizing site identified by conventional serology [13], [14]. It is not known why such BCRC neutralization sites have escaped detection before. One possible explanation is that in response to infection or immunization, the antibodies produced against these BCRC neutralization sites have been masked by those produced against the dominant antigenic determinant locating to the head of the HA molecule. The monoclonal antibodies generated against both of the BCRC neutralization sites were nevertheless found to effectively inhibit virus mediated hemeagglutination and neutralize infectivity of the virus and some of them are tested and also found to be efficacious in treatment of the respective infection even at relatively late stages of the illness [6]. This shows that the respective epitopes of the BCRC monoclonal antibodies are potential targets for broad spectrum immune intervention of influenza.

The H5 cross reacting neutralizing site is identified by a panel of monoclonal antibodies, which are exclusively reactive against the H5N1 influenza virus, but not also against other influenza virus subtypes [6], [15]. The antibodies are cross-reacting, mutually blocking binding of one another to the virus [6]. This suggests that the respective epitopes are located in proximity of one another in a single neutralizing site. The nature of the epitope of one of these antibodies, 8H5, was investigated using 12mer peptide mimics. The results suggest that 4 amino acid residues (L2,T4,L5,T9) are essential for binding with 8H5 and since these residues are located separately on the peptides recognition of which by the antibody probably depends on the juxtaposition of these residues on the peptides [16].

Presently, one of the most reactive of the peptides, p125, was randomly substituted. The amino sequences of the resulting reactive peptides were analyzed to further investigate the nature of the native epitope. The most reactive of the derived peptides was evaluated for use as a surrogate of the natural 8H5 epitope to detect antibody against the broad spectrum H5N1 neutralizing site.

## Materials and Methods

### Virus strains and antibodies

Thirteen murine monoclonal antibodies were used: 8H5,8G9,13D4, 2F2, and 3G4 were raised against H5N1 virus strains Chicken/HK/YU22/2002 (clade 8) [6]; 8C11,16D7,and 8H3, against a recombinant structural protein of Hepatitis E Virus [17]; 9B2 and 6D6 against HCV; 18B6,11H10,and 28F10, against HBV core protein. The H5N1 virus strains, Chicken/HK/YU22/2002 and Shenzhen/406H/2006, were provided by the State Key Laboratories of Emerging Infectious Diseases of the Department of Microbiology of the University of Hong Kong.

### Sub-library Construction

The sub-libraries were constructed on the phage vector M13KE (NEB Ph.D. system, New England Biolabs, Ipswich, Massachusetts, USA) according to the standard cloning techniques and M13 manipulation [18]. Oligonucleotide with amino acid substitutions of different residues of the 12mer peptide, p125, was designed and synthesized. The universal extension primer was annealed and extended with library oligonucleotide, and the resulting duplex was inserted between Acc65I and EagI sites of the vector M13KE. Sub-libraries were generated after electroporating into the bacterial strain ER2738.

### Affinity Selection of 8H5 mAb binding peptides

Affinity selection of 8H5 mAb binding peptides was performed according to the previous method [15]. Briefly, an aliquot of 1 µl of the 12-mer peptide sub-library (containing 2.9×10^11^ peptide bound phages) was pre-mixed with 30 ug 8H5 mAb in 200 ul Tris buffer saline (TBS) with 0.1%Tween-20 for 20 min at room temperature. Then, 15 ul protein A in blocking buffer (0.1 M NaHCO3 (pH 8.6) and 5 mg/ml BSA, 0.02% NaN_3_) were added to the solution and left to stand for 20 min.The unbound phage particles were removed and the bound phages were eluted. After three rounds of screening, the phage clones were analyzed by phage ELISA and DNA sequencing [16].

### Affinity determination

The kinetics of the interaction between 8H5 mAb and peptides were measured by BIAcore X1000 (BIAcore, Uppsala, Sweden). Gold-coated CM-5 sensor chips were coated with a carboxylated dextran polymer matrix with which the protein A was amine coupled. The first flow cell on the chip was coated with 2800 resonance units (RU) of the protein A, while the other flow cell was left uncoated and blocked as control. Affinity measurements were initiated by injecting 8H5 mAb at the concentration of 19 µg/ml. Then, peptide was added and allowed to bind at 20 µl/min for 2 min. The complex of 8H5 mAb and peptide was eluted by 10 mM HCl for 2 min. Each peptide was tested at five concentrations ranging from 75.8 nm to 758 nM. Curves were generated with BIA evaluation 2.1 software using kinetics simultaneous ka/kd protocols from which the equilibrium dissociation constant (KD) was calculated.

### Peptide competing to H5N1 virus

ELISA plates were coated with 2F2 and 3G4 mAbs (2 ug/ml respectively). H5N1 virus Chicken/HongKong/YU22/2002 (16HA) or Shenzhen/406H/2006 (16HA) was added to the well and incubated at 37°C for 1 h. Then the diluted peptide from 50 µg to 0.1 µg and 8H5-HRP (1∶500) were simultaneously added to the well and incubated at 37°C for 1 h. HBcAg synthetic peptide G1 was used as negative control. TMB (3,3′,5,5′-Tetramethylbenzidine) was then added and color intensity was measured in a microplate reader.

### Mimotope ELISA

Two copies of V-1b were inserted to the positions 79 and 80 of the 1-149 fragment of HBc protein in tandem with the flexible linker, GGGGS Gene coding for the fusion protein was cloned into the plasmid pTO-T7 and fusion protein HBc-V-1b was expressed and purified as described before [19]. The purified fusion protein was coated on microplate at the concentration of 5 µg/ml. Microplates similarly coated with the carrier HBV core (HBc) protein was used as negative control.

In the assay of binding specificity with 8H5 mAb, 8H5 mAb was added to test the reaction with the fusion protein. Other 3 H5-related mAbs, 3 anti-HEV mAbs, 2 anti-HCV mAbs were used as negative control. The 8H5 epitope specific ELISA was further applied to chicken serum. Chicken serum was diluted at 1∶500 and then added to microplate. Rabbit anti-chicken antibody (IgG) conjugated with HRP (Keygen, China) diluted at 1∶10000 was used as the secondary antibody.

## Results

### Random substitution of p125

To improve the mimicry of the natural 8H5 epitope by p125, we generated in succession 5 sub libraries by random substitution of 3 to 5 residues of the peptide (Table 1). Residues which were found to exhibit restricted substitutions in one sub library were specifically substituted with the same amino acids in generating the subsequent libraries. For example, substitution of residue 3 was found in the first library (12MH1) to be restricted to P,A,Q and E and this residue was specifically substituted with the same four aa in generating the subsequent sub libraries. The diversity of the 5 libraries was similar to the respective theoretical values. Screening of these libraries yielded 322 phage clones reactive with 8H5. Sequencing revealed that these reactive clones encompass 133 distinct 12mer peptides.

**Table 1:** Generation of p125 phage sub-libraries.

Phage Libraries	Random substitution§	Specific Substitutions*		Library Diversity		8H5 reactive phageΔ	
	residues	residues*	amino acids	Theoretical	Actual	Total	unique aa sequences
12MH I	3, 5, 7, 10			1.6×10^5^	1.0×10^6^	48	10
12MH II	4, 6, 8, 10	3	P, A, Q, E	2.56×10^6^	4.5×10^6^	81	14
12MH III	2, 4, 6, 8	3	P, A, Q, E	5.12×10^6^	6.5×10^6^	60	16
		10	K, R				
12MH IV	6, 9, 11	1	D, E,Q	1.02×10^6^	6.5×10^6^	94	77
		3	P, A, Q, E				
		8	A, G				
		10	K, R				
12MH V	1, 3, 6, 7, 12	8	A, G	1.28×10^7^	1.5×10^7^	39	16
		10	K, R				
Total						322	133

§ *different residues of the 12mer p125 were randomly substituted with 20 aa to generate the successive* phage libaries.

*In addition, residues found to exhibit restricted substitution were specifically substituted in generation of the subsequent libraries.

Δ 8H5 reactive phage clones were identified; the peptides were sequenced.

Table 2 summarizes amino acid substitutions observed among the 133 distinct p125-related 12mer peptides. Confirming previous findings [16], the results show that 4 residues (T2,L4,T5,L9) of the parental p125 were preserved among all the derived peptides, suggesting that these residues are essential for binding with 8H5. Another 4 residues, namely residues D1, P3, A8,and R10, exhibit restricted substitution, suggesting that these residues may affect 8H5 binding. The remaining 4 residues, T6, A7, L11, V12, could be randomly substituted, suggesting that they may not contribute to 8H5 binding or minimally so. Thus, the residues involved in binding are interspersed among residues that apparently do not participate in binding, suggesting that 8H5 binds to discontinuous residues on the peptides.

**Table 2:** Amino acid substitution of 8H5-reactive 12mer peptides.

Residues	p125	p125 related peptides (n = 133)
1	D	D,E,Q
2	T	T
3	P	P,A,Q,E
4	L	L
5	T	T
6	T	20 natural aa
7	A	20 natural aa
8	A	A,G
9	L	L
10	R	K, R
11	L	20 natural aa
12	V	14 natural aa

*133 distinct 12mer peptides reactive with 8H5 were identified from 5 sub-libraries of p125 as described in* *Table 1* *.*

### Effects of amino acid substitutions on 8H5 binding

**Figure 1:**
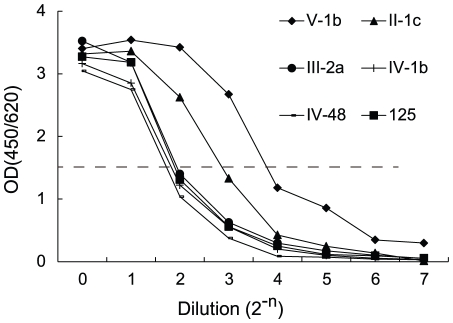
Titration of phage displaying p125 and related 12mer peptides. 10^13^ phage displaying the p125 or the related 12mer peptides, V-1b, II-1c, III-2a, IV-1b, and IV-48 was serially diluted and allowed to react with microplates previously coated with 8H5 for 30 min. The plates were in turn reacted with anti-phage. The 8H5 binding activity of the phage is determined from the titration curves and expressed as the phage dilution yielding an OD value of 1.5 (T_OD1.5_).

The 8H5 reactivity of the phages displaying p125 and 133 different related 12mer peptides was determined by titration using microplates previously coated with 8H5 (see Fig. 1). The reactivity was expressed in 8H5 titer, defined as the phage dilution yielding an OD value of 1.5 (T_O D 1.5_). Compared with the 8H5 titer of parental p125 phage (T_O D 1.5_ = 2.96), the 8H5 reactivity of three phage clones displaying the peptides III-2a, II-1c and V-1b, respectively, was variously increased, with 8H5 titers of 3.27, 7.07 and 13.23, respectively (Table 3). The 8H5 titers of the other 130 phage clones was either comparable to that of the parental p125 phage, as exemplified by the phage bearing the peptide IV-1b (T_O D 1.5_ = 2.76), or lower with T_O D 1.5_<2.0 (not shown).

**Table 3:** 8H5 reactivity and dissociation constants of p125 and related 12mer peptides.

Peptide	Amino acid substitutions†	Titer‡	Blocking Dose*		Kd§
		(T_od1.5_)	Yu22	SZ/406H	(MOLAR)
p125		2.96	18.34 ug	11.16 ug	8.24E-07
IV-1b	D1E, P3E,R10K (T6I, L11Y)	2.76	>50 ug	>50 ug	9.80E-07
III-2a	P3E (T6K, A7Q)	3.27	15.19 ug	6.10 ug	2.84E-08
II-1c	A8G, R10K (T6I)	7.07	8.90 ug	2.38 ug	1.15E-08
V-1b	D1E, A8G, R10K (T6I, V12K)	13.23	5.70 ug	2.31 ug	3.16E-09

† Residues which could be randomly substituted shown in parenthesis.

‡ *The 8H5 reactivity of phage bearing p125 and the related 12mer peptides was determined by titration as in* *Figure 1* *and expressed in titer defined as the phage dilution yielding an OD value of 1.5 (T_od1.5_).*

**Figure 2:**
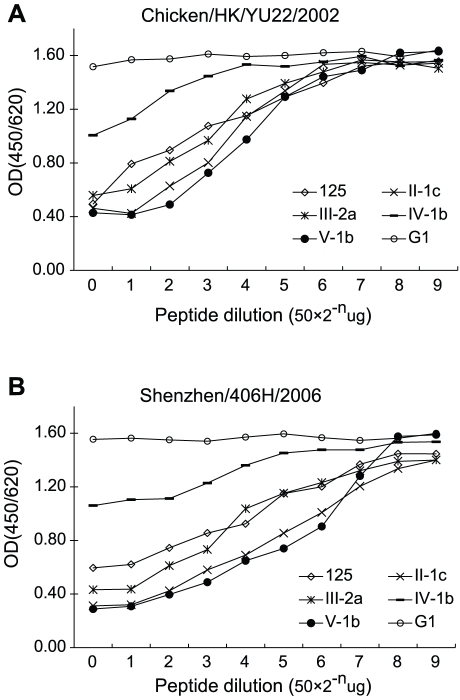
Titration of synthetic 12mer peptides. Serially diluted aliquots containing indicated amounts of synthetic peptide or HBcAg synthetic peptide (G1) were allowed to react with 8H5. The residual antibody available for virus binding with 2 strains of H5N1 was determined by ELISA. 8H5 blocking activity of peptides is expressed as blocking dose, defined as the amount of peptide that reduces 8H5 binding by 50%.

*8H5 reactivity of the respective peptides was determined as in *Figure 2* *and expressed in blocking dose, defined as amount of the peptide that block binding of 8H5 to the indicated strain of the H5N1 avian influenza virus by 50%.*

§ *Affinity of the peptides for 8H5 was determined by surface plasma resonance using BiaCore and expressed as dissociation constant (Kd).*

The 8H5 reactivity of the 5 peptides was further assessed by their capacity to block 8H5 from binding with two strains of H5N1 influenza virus. In these experiments, serially diluted aliquots of the synthetic peptides were allowed to react with 8H5 and the residual antibody available for binding with the viruses was determined by ELISA (Fig. 2). 8H5 reactivity of the peptides was expressed in 8H5 blocking dose, which is defined as the amount of the peptide that blocks 8H5 binding by 50%.

The affinity of the peptides for 8H5 was also determined by surface plasma resonance using BiaCORE. Table 3 relates the amino acid substitutions of the p125 related peptides with their respective 8H5 reactivity and affinity for the antibody. The results show that V-1b is the most reactive with 8H5 and has the highest affinity for the antibody and suggest that this is probably attributed to the substitutions, D1E, A8G and R10K. The peptide also contains two other substitutions, T6I and V12K, but since both residues could be randomly substituted, they are unlikely to affect the 8H5 reactivity of the peptide or its affinity for the antibody. Two other peptides, II-1c and III-2a, exhibit a moderate increase in 8H5 reactivity and affinity for the antibody and this is probably attributed to A8G and R10K, in the case of the peptide II-1c, and to P3E, in the case of the III-2a peptide. On the other hand, the 8H5 reactivity of IV-1b and its affinity for the antibody was lower than the parental p125 and this is probably attributed to D1E, P3E and R10K. II-1c, III-2a and IV-1b also variously contain substitutions at residues T6, A7 L11 and V12, which are considered unlikely to affect 8H5 binding.

### 8H5 epitope specific ELISA

**Figure 3:**
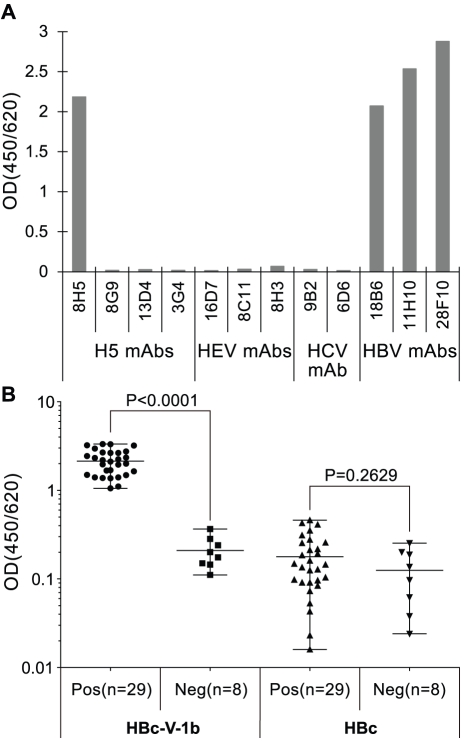
Evaluation of ELISA produced with the 8H5 reactive12mer peptide (V-1b). In the top panel, microplates were coated with V-1b-HB core antigen (HBc-V-1b) were reacted with monoclonal antibodies specific for H5 molecule of H5N1 avian influenza virus (H5 mAbs), hepatitis E virus (HEV mAbs), hepatitis C virus (HCV mAb) and monoclonal antibodies specific for the HBV core antigen (HBV mAbs). In the bottom panel, these and also microplates coated with HBc alone were reacted with serum samples from 29 chicken infected with the H5N1 avian influenza virus and 8 control uninfected animals.

An enzyme-linked immunosorbant assay (ELISA) was produced with V-1b fused to the hepatitis B virus core antigen (HBcAg). The antigenic specificity of the assay was assessed against 8H5, other control monoclonal antibodies and serum samples from chicken previously infected or not with H5N1 virus (Fig. 3). The results show that the assay is exclusively specific for 8H5. Apart from the monoclonal antibodies generated against the carrier protein, the assay exclusively binds 8H5, but not also the other related broad spectrum H5N1 avian influenza neutralizing antibodies or the unrelated control antibodies raised against HEV and HCV, respectively (upper panel). The chicken serum samples were obtained from a poultry farm in southern China and included 29 which were previously tested to be reactive for H5N1 avian influenza virus and 8 which are seronegative for the virus (Chen Y, PhD thesis 2010, Xiamen University, China). The results show that only the H5N1 avian influenza reactive samples were also 8H5 reactive (lower panel).

## Discussion

We have derived a 12mer peptide, V1-b, from phage display libraries, which closely mimics the natural epitope of the broad spectrum H5N1 avian virus neutralizing monoclonal antibody, 8H5. ELISA produced with this peptide is specific for the antibody and serves to distinguish between serum samples from chicken which had and had not been previously infected with H5N1 avian Influenza virus. This suggests that this peptide could be used as a surrogate of the natural 8H5 epitope to detect antibody against the broad spectrum neutralization site of the H5N1 avian influenza virus in serum samples.

We generated in succession 5 phage display libraries by systematically substituting different residues of p125 and analyzing the aa sequences of 133 8H5 reactive peptides thus derived. Involvement of each residue in binding with the antibody was assessed according to its susceptibility to substitution. Confirming earlier findings [6] the residues T2,L4,T5,L9 of the parental p125 were considered to be essential for antibody binding, because they were conserved among all the reactive peptides. It was further shown that these residues were interspersed among 4 other residues (D1, P3, A8, R10), which may contribute to antibody binding, because they exhibited restricted patterns of substitution and another 4 (T6, A7, L11, V12), which may not involved in antibody binding, because they could be randomly substituted.

Titration of 8H5 binding activity found that the most of the peptides were either less or similarly reactive as the parental p125, except for 3 which are more reactive than p125. The peptide V1-b is the most reactive of the latter, showing the highest level of 8H5 binding and capacity to block the antibody from binding with H5N1 avian influenzavirus. The affinity of this peptide for 8H5 was found by plasma surface resonance to be 3.16×10^9^ M, 38 time that of the parental p125. ELISA produced with this peptide shows that it binds exclusively with 8H5 and not also the cross-reacting broad spectrum H5N1 neutralizing antibodies The peptide was reactive with serum samples from chickens infected with H5N1 avian influenza virus, but not also the samples from control uninfected animal. Consistent with findings by co-crystalization of a heterosubtypic antibody and HA molecule [6], V1-b was aligned to amino acid residues 44, 77, 267, 268, 277, 281, 297–302 locating to the stem of the HA molecule, using the Pepsurf software (http://pepitope.tau.ac.il/).

Peptide mimics have been used as surrogates of the natural epitopes for diagnosis [20], [21], [22] and vaccines [23], [24], [25] and treatment [23], [26], [27]. The present findings show that the 12 mer peptide, V1-b, closely mimics the natural 8H5 epitope, to the extent that it could be used as surrogate of the natural epitope in an objective ELISA assay to detect antibody against the broad spectrum H5N1 avian Influenza virus neutralization site in serum samples.
